# Deciphering the Structural and Functional Effects of the R1150W Non-Synonymous Variant in SCN9A Linked to Altered Pain Perception

**DOI:** 10.3390/neurosci6020038

**Published:** 2025-05-02

**Authors:** Faisal A. Al-Allaf, Zainularifeen Abduljaleel, Mohammad Athar

**Affiliations:** 1 Department of Medical Genetics, Faculty of Medicine, Umm Al-Qura University, Makkah 21955, Saudi Arabia; zainulbio@gmail.com; 2 Science and Technology Unit, Umm Al-Qura University, Makkah 21955, Saudi Arabia

**Keywords:** SCN9A, pain perception, structural biology, computational modeling, pain modulation, congenital insensitivity to pain

## Abstract

The SCN9A gene, a critical regulator of pain perception, encodes the voltage-gated sodium channel Nav1.7, a key mediator of pain signal transmission. This study conducts a multimodal assessment of SCN9A, integrating genetic variation, structural architecture, and molecular dynamics to elucidate its role in pain regulation. Using advanced computational methods, I-TASSER simulations generated structural decoys of the SCN9A homology domain, producing an ensemble of conformational states. SPICKER clustering identified five representative models with a C-score of −3.19 and TM-score of 0.36 ± 0.12, reflecting moderate structural similarity to experimental templates while highlighting deviations that may underpin functional divergence. Validation via ProSA-web supported model reliability, yielding a Z-score of −1.63, consistent with native-like structures. Central to the analysis was the R1150W non-synonymous variant, a potential pathogenic variant. Structural modeling revealed localized stability in the mutant conformation but disrupted hydrogen bonding and altered charge distribution. Its pathogenicity was underscored by a high MetaRNN score (0.7978498) and proximity to evolutionarily conserved regions, suggesting functional importance. Notably, the variant lies within the Sodium-Ion-Transport-Associated Domain, where perturbations could impair ion conductance and channel gating—mechanisms critical for neuronal excitability. These findings illuminate how SCN9A variants disrupt pain signaling, linking genetic anomalies to molecular dysfunction. While computational insights advance mechanistic understanding, experimental validation is essential to confirm the variant’s impact on Nav1.7 dynamics and cellular physiology. By refining SCN9A’s molecular blueprint and highlighting its therapeutic potential as a target for precision analgesics, this work provides a roadmap for mitigating pain-related disorders through channel-specific modulation. Integrating structural bioinformatics with functional genomics, this study deciphers SCN9A’s role in pain biology, laying the groundwork for novel strategies to manage pathological pain.

## 1. Introduction

The complex interplay of nociception and pain perception supports the delicate biological machinery that is necessary for the survival of the organism. Nociception is a highly sensitive sensing modality that has evolved to detect a wide range of noxious stimuli from damaging mechanical traumas to damaging thermal extremes and respond quickly to prevent further damage. Pain perception, which arises as the end-point of all this sensory integration, acts as an imperative alarm mechanism, signifying a potential threat to the organism and initiating protective responses necessary for tissue repair and maintenance of homeostasis [1].

However, the evolutionary benefits of pain mechanisms are juxtaposed with the burdens of chronic pain conditions that afflict millions worldwide. Chronic pain, often arising from maladaptive changes in nociceptive processing following tissue injury or disease, poses significant challenges to both individuals and healthcare systems [2]. The debilitating nature of chronic pain not only impairs quality of life but also imposes substantial socioeconomic burdens through healthcare expenditures and lost productivity [3].

In this context, the quest for effective pain management strategies has been a longstanding endeavor, driven by the imperative to alleviate suffering and restore functionality to those afflicted. Despite advances in pharmacotherapy and interventional techniques, many individuals with chronic pain continue to experience inadequate relief or intolerable side-effects from existing treatments [4]. This treatment gap underscores the need for novel therapeutic approaches that address the diverse etiologies and manifestations of chronic pain while minimizing adverse effects and optimizing patient outcomes.

The SCN9A gene, encoding the Nav1.7 voltage-gated sodium channel, occupies a central position in the molecular landscape of nociception and pain modulation. Nav1.7, predominantly expressed in peripheral sensory neurons, governs the generation and propagation of action potentials critical for nociceptive signaling [5]. Through its role in regulating neuronal excitability, SCN9A exerts precise control over pain thresholds and sensory perception, making it a prime target for investigating pain pathophysiology and developing targeted therapies [6].

SCN9A comprises 26 exons and encodes a protein of 1977 amino acids, forming four homologous domains (DI-DIV), each consisting of six transmembrane segments (S1–S6). The structural organization of Nav1.7 is essential for its function, including voltage sensing and ion conduction. Variants in SCN9A can lead to either gain-of-function or loss-of-function effects, influencing the excitability of sensory neurons and thereby altering pain perception [5].

A wide array of disease-causing variants in SCN9A have been identified, contributing to distinct clinical phenotypes. Gain-of-function mutations are commonly associated with conditions such as primary erythromelalgia (PEM) and paroxysmal extreme pain disorder (PEPD), characterized by episodes of severe pain triggered by temperature or mechanical stimuli [7]. Conversely, loss-of-function mutations result in congenital insensitivity to pain (CIP), a rare disorder where affected individuals are unable to perceive pain despite otherwise normal sensory modalities [8,9]. This diverse phenotypic spectrum underscores the pivotal role of SCN9A in human pain physiology and the potential of its variants to inform personalized pain therapies.

The genetic and functional diversity inherent in SCN9A variants underscores the complexity of pain phenotypes observed in clinical practice. From inherited disorders of pain insensitivity to debilitating neuropathic pain syndromes, aberrations in SCN9A function can manifest across a broad spectrum of clinical presentations [10]. Understanding the mechanistic underpinnings of SCN9A-associated pain disorders offers valuable insights into the physiological mechanisms governing pain perception and opens avenues for precision medicine approaches tailored to individual patients’ genetic profiles [11].

One notable SCN9A variant is R1150W (rs6746030), which has been the focus of multiple studies due to its intriguing role in modulating pain sensitivity. This single nucleotide polymorphism leads to an amino acid change in the intracellular loop between domains II and III of the Nav1.7 protein—a region critical for proper gating and modulation of the channel’s activity. The substitution of arginine (a positively charged, polar residue) with tryptophan (a bulky, nonpolar residue) may alter protein conformation and disrupt the electrostatic environment essential for ion conduction and voltage sensing [12]. Although it was initially identified as a causal variant for primary erythromelalgia, subsequent findings revealed a minor allele (A) frequency of approximately 10%, leading to the re-evaluation of its pathogenicity. Interestingly, the R1150W variant has been significantly associated with heightened pain perception across various conditions, including osteoarthritis, sciatica, phantom limb pain, lumbar discectomy, and pancreatitis [13]. Further research made a similar link; for instance, Faber et al. (2012) found that carriers of the R1150W variant rated their pain higher in cold pressor and heat pain tests [14]. The functional consequences of this variant on Nav1.7 activity and its potential role in modifying pain susceptibility are not well understood. Electrophysiological studies demonstrated that the minor A allele enhances Nav1.7 activity, thereby increasing neuronal excitability and lowering pain thresholds [12]. Additionally, the variant was found to confer protection against severe oxaliplatin-induced peripheral neuropathy [15] and has been linked to increased preoperative pain intensity in lumbar disc herniation patients, without influencing postoperative recovery [16]. Recently, Leznicka et al. (2023) examined the association between the R1150W variant and pain perception in combat athletes and non-athletes, suggesting a potential role of this variant in modulating pain tolerance [17]. These findings provided biological plausibility for the role of this genetic variant in modulating pain sensitivity. However, conflicting evidence from a cohort study indicated that two patients with congenital insensitivity to pain (CIP) carried the minor allele, challenging the straightforward interpretation of its role [8]. This paradox suggests that R1150W may not be solely sufficient in determining phenotype, and its effect may depend on modifying factors, haplotype background, or epistatic interactions. These findings highlight the complex, context-dependent effects of R1150W on pain phenotypes and underscore the necessity for further investigation into its mechanistic impact on Nav1.7 function.

As researchers delve deeper into the molecular intricacies of SCN9A and its interactions within the broader pain signaling network, the promise of targeted therapeutic interventions looms ever closer. By elucidating the genetic determinants, structural dynamics, and functional consequences of SCN9A variants, investigators aim to unravel the intricate web of pain modulation and pave the way for precision therapies that alleviate suffering with unprecedented efficacy and precision. In this endeavor, collaborative efforts across disciplines and innovative research methodologies will be instrumental in translating basic science discoveries into tangible clinical benefits for patients grappling with the burden of chronic pain.

The results here intend to serve as a deep dive into the 3D structure prediction algorithm SPICKER and homology modeling. On top of that, the pipeline created to analyze these structures can be readily used to determine the quality of the predicted structure from this algorithm. In the end, we suggest steps to increase the quality of the predicted structures with various in silico methods. This information aims to provide valuable insights into the development of targeted therapeutic interventions for chronic pain conditions associated with SCN9A variants, ultimately contributing to the advancement of precision medicine approaches in pain management.

## 2. Material and Methods

### 2.1. Decoy Ensemble Generation and Model Selection

**Figure 1:**
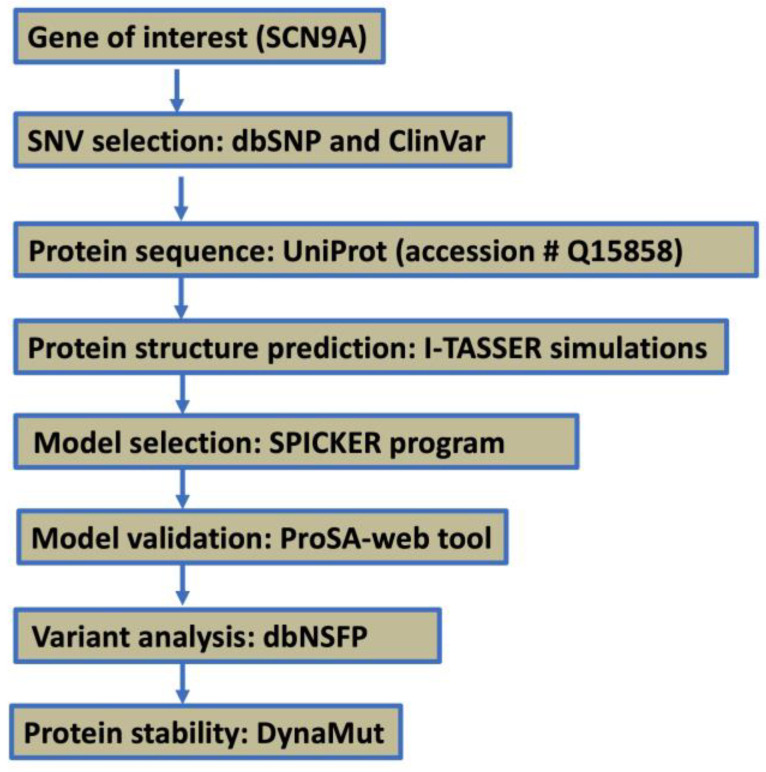
A step-by-step workflow diagram.

The SCN9A gene variant R1150W (rs6746030) was selected for computational analysis based on its reported association with altered pain sensitivity and electrophysiological effects. The step-by-step workflow is illustrated in Figure 1. The variant was retrieved from the dbSNP and ClinVar databases, while the reference protein sequence for Nav1.7 was obtained from UniProt (Accession ID: Q15858).

I-TASSER simulations [18,19,20] were utilized to generate an extensive ensemble of structural conformations (decoys) for the SCN9A homology protein domain, consisting of 205 amino acid residues. I-TASSER is a hierarchical protein structure prediction tool that generates 3D models based on sequence homology and ab initio modeling. The SPICKER program [21] was employed to cluster decoys based on pair-wise structure similarity, resulting in up to five models corresponding to the five largest structure clusters. SPICKER is a clustering algorithm designed to identify near-native models from a set of predicted protein structure decoys. Quantitative confidence assessment was conducted using the C-score, which ranged from −5 to 2, with higher values indicating higher confidence. Models were selected based on C-score assessment, TM-score, and RMSD values of experimentally determined structures.

### 2.2. Structural Validation of SCN9A Homology Protein Domain

The structural integrity of the SCN9A homology protein domain was rigorously validated using the ProSA-web tool, which assesses model quality based on energy profile analysis [22]. A Z-score analysis was performed to assess model reliability by comparing it with experimentally determined structures available in the Protein Data Bank (PDB) through X-ray analysis. The obtained Z-score fell within an acceptable range, confirming the reliability and accuracy of the computational model. Additionally, TM-score and RMSD values were utilized to evaluate the structural similarity to and deviation from experimentally determined structures.

### 2.3. Local Stability and Features of R1150W Substitution

Our investigation into the structural consequences of the SCN9A gene variant, specifically the R1150W substitution, utilized a comprehensive approach. We analyzed various structural parameters, including secondary structure, charge distribution, hydrogen bonding patterns, and cavity volume, to understand how the variant impacts protein stability and functionality within the context of pain modulation. To further enhance our analysis, we employed Missense3D [23] and the Phyre2 algorithm [24], which allowed us to assess the structural impact of missense variants and gain deeper insights into the effects of variants on protein structure and function. Additionally, we utilized DynaMut, integrating graph-based signatures with normal mode dynamics, to generate a consensus prediction of the variant’s impact on protein stability. Our approach demonstrated superior performance compared to alternative methods, achieving a correlation of up to 0.70 on blind tests (*p*-value < 0.001). Furthermore, DynaMut [25] suggestions a comprehensive suite for protein motion and flexibility analysis, enhancing our understanding of the structural implications of genetic variations. This integrated approach provides valuable insights into the structural biology of SCN9A variants, offering a deeper understanding of their functional implications in pain sensitivity and modulation.

### 2.4. Functional Implications of SCN9A Variant

Functional ramifications of the SCN9A protein variant, particularly the substitution of arginine with tryptophan at position 1150, were investigated. Variant analysis using dbNSFP was performed to assess pathogenicity likelihood. Conservation analysis and domain-specific investigations were conducted to elucidate the variant’s impact on essential protein functions and folding dynamics. Furthermore, the HOPE program [26] was utilized to predict the potential structural and functional consequences of the variant. HOPE, a fully automatic program, collects information from various sources, including 3D protein coordinates, sequence annotations from UniProt, and predictions by DAS services [27]. Homology models are constructed using YASARA [28], and the collected data are utilized in a decision scheme to identify the effects of the variant on the protein’s 3D structure and function. HOPE generates a comprehensive report with text, figures, and animations, facilitating understanding for biomedical researchers.

## 3. Results

### 3.1. Structural Prediction and Confidence Assessment of the SCN9A Homology Domain

The protein structure prediction uses I-TASSER simulations to generate an extensive ensemble of structural conformations known as decoys for each target. Specifically focusing on the SCN9A homology protein domain, encompassing 205 residues, the final model selection employs the SPICKER program to cluster decoys based on pair-wise structure similarity, resulting in up to five models corresponding to the five largest structure clusters. Quantitative confidence assessment is achieved through the C-score, calculated based on the significance of threading template alignments and the convergence parameters of structure assembly simulations. The C-score, ranging from −5 to 2, serves as a confidence indicator; for our SCN9A model, the computed C-score is −3.19. Additionally, the estimated TM-score is 0.36 ± 0.12, providing insights into structural similarity, while the estimated RMSD is 12.9 ± 4.2 Å, indicating the degree of deviation from the experimentally determined structure. These results contribute to a comprehensive understanding of the quality and reliability of the predicted SCN9A homology protein domain models. TM-score and RMSD values align with observed correlations and provide valuable metrics for assessing the accuracy of the predicted structures. While typically ranked by cluster size, lower-rank models may exhibit higher C-scores in rare cases, as demonstrated in benchmark tests. In instances of well-converged simulations, generating fewer than five clusters suggests high-quality models due to successful convergence in the simulation process.

### 3.2. Structural Validation and Functional Insights of the SCN9A Homology Protein Domain

**Figure 2:**
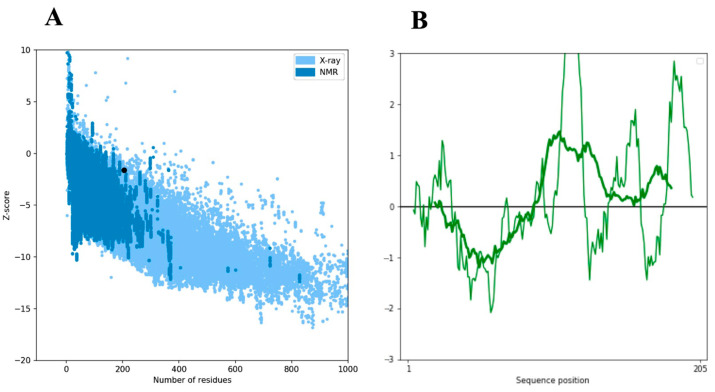
The ProSA-web z-score plot for the SCN9A domain structure. (**A**) The model’s z-score of −1.63 falls within the typical range of conformational parameters for native proteins, as depicted by the black dot on the plot. The ProSA-web analysis integrates data from protein experimental chains in the PDB determined by nuclear magnetic resonance (NMR) (dark blue) and X-ray crystallography (light blue). (**B**) The Z-score of the modeled SCN9A domain structure is highlighted as large dots, and the right graph depicts the energy plot of the SCN9A model.

We conducted a comprehensive exploration of the SCN9A homology protein domain, encompassing 205 residues, utilizing the powerful ProSA-web tool. Our aim was to rigorously validate the structural integrity of this region by comparing it with experimentally determined structures available in the Protein Data Bank (PDB) through X-ray analysis. SCN9A, a protein prominently expressed in nociceptive primary sensory neurons, plays a pivotal role in amplifying small depolarizations. The application of ProSA-web for structural validation yielded a Z-score of −1.63, attesting to the reliability and accuracy of our determined model (Figure 2A,B). This score falls within an acceptable range, reinforcing the confidence in the fidelity of our structural predictions. Our findings not only validate the computational model but also contribute to the broader understanding of SCN9A’s structural intricacies. It is crucial to underscore the physiological significance of SCN9A, particularly in its preferential expression within nociceptive primary sensory neurons. The amplification of small depolarizations by SCN9A underscores its role in modulating neuronal excitability and sensitivity to pain stimuli. Moreover, our newly determined structure offers unique insights into the theatrical aspects of the SCN9A protein. The intricacies revealed by our analysis contribute to a more nuanced comprehension of the functional implications of SCN9A in cellular processes. By bridging computational modeling with experimental validation, this study adds a valuable layer to the existing body of knowledge regarding SCN9A’s structural biology. The energy plot illustrates the quality of the theoretical model, highlighting potential issues with positive values. To enhance clarity, we smoothed the plot by averaging energies over 40-residue fragments, assigning the result to the central residue at position i + 19. Overall, our meticulous application of ProSA-web, coupled with the scrutiny of X-ray-derived structures, establishes a robust foundation for understanding the SCN9A homology protein domain. The insights gained from our analysis not only affirm the validity of our model but also deepen our understanding of the structural and functional attributes of SCN9A, offering potential avenues for further investigation and therapeutic exploration.

### 3.3. Structural Consequences of the R1150W Variant in the SCN9A Gene

**Figure 3:**
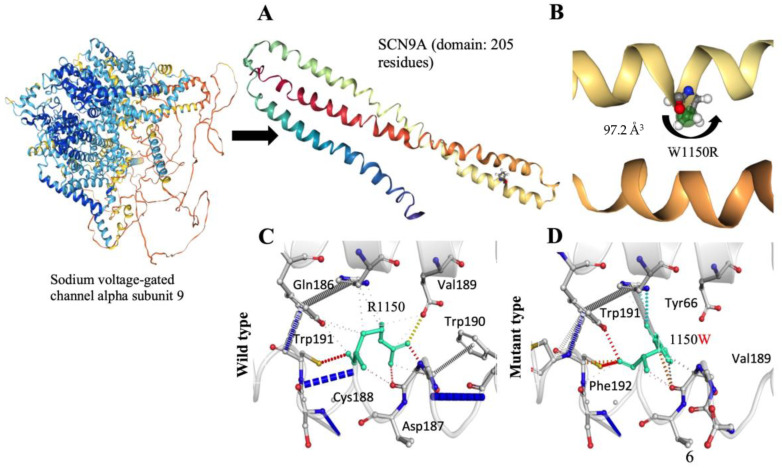
(**A**) The ab initio protein modeling structure of the SCN9A protein domain, consisting of 205 amino acid residues. (**B**) The substitution leads to a notable reduction in the cavity volume, estimated at 97.2 Å^3^. (**C**) Wild-type residues (R1150), depicted in light green, are shown as sticks along with neighboring residues involved in various interactions. (**D**) Mutant-type residues (1150W), also depicted in light green, are represented as sticks alongside neighboring residues engaged in interactions.

The meticulous examination of the structural consequences of the SCN9A gene variant, specifically the R1150W substitution, reveals distinctive features associated with pain perception. Notably, the absence of a CYS residue in the wild-type structure excludes the possibility of disulfide bond formation. The introduced R1150W substitution does not give rise to a proline or triggers clash alerts, suggesting stability in the local structural context. Crucially, the R1150W variant does not replace a buried hydrophobic residue with a hydrophilic one, nor does it prompt alerts related to buried charged or uncharged residues. The secondary structure, identified as a bend (‘S’), remains unaltered, indicating that the R1150W variant does not compromise the fundamental structural motifs of the protein. Examining the charge distribution, the R1150W variant does not induce a buried charge switch, and the alteration from a positively charged wild-type residue to an uncharged mutant residue occurs without violating phi/psi angle preferences. Furthermore, there is no replacement of a buried charged residue with an uncharged one, and buried glycine substitution is absent. The hydrogen bond analysis reveals that the R1150W variant does not cause a complete disruption of side-chain or main-chain hydrogen bonds, and the wild-type residue is not buried. The intricate landscape of salt bridges is explored, with a detected disruption of a wild-type salt bridge (Figure 3A,B). Importantly, despite this disruption, the wild-type residue is not buried, maintaining a certain level of structural integrity. Intriguingly, the R1150W variant leads to the contraction of the cavity volume by 97.2 Å^3^, indicating a localized structural modification within the protein (Figure 3B–D). The transition from an exposed wild-type residue to an exposed mutant residue is observed, adding a layer of complexity to the structural changes induced by the R1150W variant. Critically, there is no cis proline replacement or the presence of glycine in a bend. These comprehensive structural insights, specifically addressing the R1150W variant, provide valuable contributions to understanding the molecular underpinnings of pain perception associated with the SCN9A gene, offering a detailed characterization of the mutational impact within the protein structure.

### 3.4. Functional Impact of SCN9A R1150W Variant on Protein Structure and Activity

Our detailed investigation into the functional ramifications of the SCN9A protein variant, specifically the substitution of arginine with tryptophan at position 1150, unfolds crucial insights in the absence of known structural information. The mutational event introduces a larger, more hydrophobic residue, instigating a shift from a positively charged wild-type residue to a neutral mutant residue. This alteration potentially engenders profound consequences for the protein’s interactive landscape, hydrogen bonding patterns, and overall folding dynamics. Employing dbNSFP for variant analysis, we uncovered a MetaRNN score of 0.7978498, signifying a notable likelihood of pathogenicity. Conservation analysis accentuates the mutant residue’s adjacency to a highly conserved position, reinforcing its presumed functional significance. Within the Sodium-Ion-Transport-Associated Domain, intricately linked to Voltage-Gated Sodium Channel Activity, the variant emerges as a potential disruptor, impacting essential protein functions. Structural consequences include the loss of charge, raising concerns about interaction disruptions, and the heightened hydrophobicity, potentially perturbing the correct folding of the protein. Collectively, these findings underscore the substantial impact of the variant on SCN9A, urging further experimental validation for a comprehensive elucidation of its intricate functional implications in cellular processes.

## 4. Discussion

**Figure 4:**
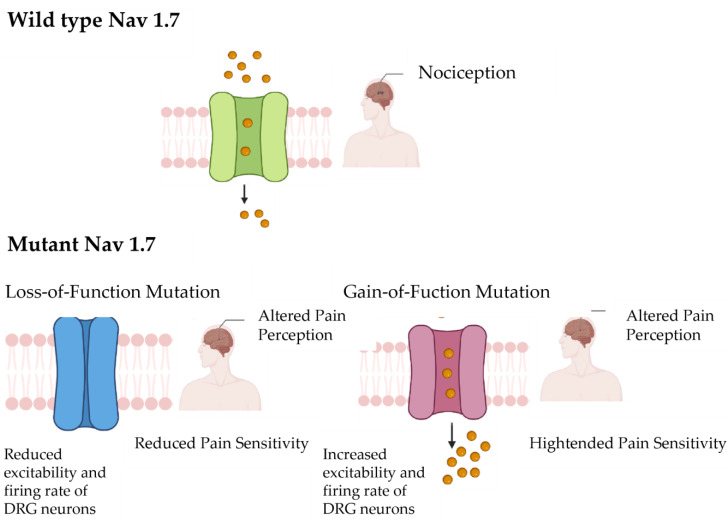
This figure illustrates the contrast between the wild-type Nav1.7 channel, which is associated with normal pain perception (nociception), and the mutant Nav1.7 channel, which leads to altered pain perception. The wild-type Nav1.7 channel functions normally, transmitting nociceptive signals in response to painful stimuli. However, a variant of the Nav1.7 channel can disrupt this process, resulting in a state of altered pain perception. This figure provides a visual representation of the functional differences between wild-type and mutant Nav1.7 channels in modulating pain perception.

The SCN9A gene is implicated in three distinct human pain disorders. In cases of nonsense variants, individuals experience a total lack of pain sensation, while activating variants lead to intense episodic pain, characteristic of paroxysmal extreme pain disorder and primary erythermalgia [13,15,29] (Figure 4). This prompted our exploration into whether variations in single nucleotide polymorphisms (SNPs) within the SCN9A gene correlate with varying levels of pain perception among the broader population. Studies have demonstrated a highly significant association (*p* = 0.0001) between the common polymorphism rs6746030 in the SCN9A gene and pain perception across various pathological conditions [13], highlighting its potential relevance in pain modulation. Among the five SCN9A SNPs initially found to be significantly associated with pain levels in osteoarthritis, only rs6746030 results in a coding change, whereas the other four are not predicted to be detrimental. The minor allele of rs6746030 occurs at a frequency of approximately 10% and is associated with increased pain perception, resulting in an amino acid change from arginine to tryptophan at position 1150 of Nav1.7 [17,30]. This residue lies in the intracellular loop between transmembrane domains II and III of Nav1.7, a region of the channel with unknown function. However, 1150R is an evolutionarily conserved amino acid, suggesting that it could contribute to the normal function of Nav1.7.

Although several studies have implicated R1150W in pain sensitivity modulation, the evidence is not entirely consistent, however. For example, a recent study by Newton et al. (2025) using the UK Biobank found no relationship between variants in SCN9A, including R1150W, and pain frequency or analgesic use [31]. These results are at odds with previous clinical and in vitro studies that had indicated a gain-of-function phenotype for this variant [12,13]. The differences may be due to population heterogeneity, phenotype definitions, sample size, ethnic mix, and gene–environment interactions.

With such conflicting data, it is unclear whether R1150W is a functional modifier, a neutral polymorphism, or a context-sensitive risk factor for pain disorders. So SCN9A may be an exciting target for new analgesic drugs, but its potential as a biomarker indicating susceptibility to pain should be interpreted with care and needs to be tested in varying populations and under different experimental conditions.

The R1150W variant of the SCN9A gene is a complex and context-sensitive polymorphism. There are some early studies and in vitro data suggesting that it has a functional role in enhancing Nav1.7 excitability and making people more pain-sensitive, but at the population level, there has not been a definitive pathogenic connection. It is thus not appropriately termed a disease-causing variant, but rather a relatively common non-synonymous variant with differing phenotypic effects. This illustrates the need to use a variety of evidential sources—functional, epidemiological, clinical phenotyping—in a more sophisticated way to assess the likely pathogenicity of common polymorphisms in pain-associated genes.

Based on these data, the molecular basis of pain perception, particularly in the context of chronic pain disorders, is critical for developing effective therapeutic interventions. Our study focused on unraveling the structural and functional implications of SCN9A variants, shedding light on the intricate mechanisms underlying altered pain perception and modulation. Currently, there is no available mutation on the specific SCN9A protein. The human Nav1.7 channel’s amino acid sequence was acquired from the UniProt database, identified under accession number Q15858. The structural predictions generated using I-TASSER simulations and validated through rigorous analysis using ProSA-web provide valuable insights into the SCN9A homology protein domain of 205 amino acid residues. Our models exhibit significant structural similarity to experimentally determined structures, confirming the reliability and accuracy of our computational approach. These findings lay a solid foundation for further investigations into the structural biology of SCN9A and its role in treating pain.

It has been demonstrated that the R1150W variant significantly alters Nav1.7 function by depolarizing activation and increasing neuronal excitability in dorsal root ganglion (DRG) neurons. The R1150W allele led to a shift in the resting membrane potential and an increase in firing frequency, suggesting a potential role in heightened pain sensitivity [12]. These results support previous reports that link R1150W to increased susceptibility to pain, reinforcing the idea that common polymorphisms in SCN9A may contribute to inter-individual differences in pain perception. While R1150W is not a pathological variant in the classical sense, its functional effects highlight the complexity of pain modulation through Nav1.7 variants. Further studies, particularly in diverse populations, are needed to explore its clinical significance and potential implications for personalized pain management strategies.

The detailed examination of the R1150W substitution within the SCN9A protein reveals novel insights into the molecular basis of pain perception. Our analysis reveals that this variant leads to localized structural modifications, including alterations in hydrogen bonding patterns and cavity volume. Importantly, the mutant residue introduces changes in charge distribution and hydrophobicity, potentially impacting protein stability and function. Structural mutants induce distortions in the protein scaffold, resulting in the formation of internal cavities or surface crevices and ultimately leading to conformational changes. The R1150W variants had significant effects on the thermostability of the protein, persisting even after substantial alterations. These structural insights deepen our understanding of the functional consequences of the SCN9A variant and provide a basis for future studies exploring their role in pain perception.

Furthermore, our investigation into the functional implications of the R1150W variant highlights its potential pathogenicity and impact on essential protein functions. Variant analysis using dbNSFP suggests a high likelihood of pathogenicity, while conservation analysis underscores the variant’s proximity to highly conserved regions crucial for SCN9A activity. The variant’s effects on charge distribution and hydrophobicity within functional domains further emphasize its significance in modulating pain sensitivity. These findings underscore the importance of considering both structural and functional aspects when evaluating the impact of SCN9A variants on pain perception.

Overall, our study provides valuable insights into the structural and functional consequences of SCN9A variants, advancing our understanding of pain modulation mechanisms. By elucidating the molecular basis of altered pain perception associated with SCN9A variants, we lay the groundwork for the development of targeted therapeutic interventions for chronic pain disorders. Future research efforts should focus on validating our findings experimentally and exploring additional SCN9A variants to comprehensively characterize their role in pain processing. Collaborative endeavors combining computational modeling, experimental validation, and clinical studies will be crucial for translating these insights into clinical applications and improving pain management strategies.

## 5. Conclusions

Our study highlights the crucial role of the SCN9A variant in modulating pain perception by altering the structural and functional properties of the Nav1.7 channel. Specifically, we demonstrate that the R1150W variant induces conformational changes that may affect protein stability, thermostability, and neuronal excitability, which may contribute to abnormal pain sensitivity. While our findings establish a structural basis for these alterations, the context-dependent effects of R1150W, including its paradoxical association with both heightened pain perception and congenital insensitivity to pain (CIP), warrant further investigation. Despite these insights, our study has limitations, including the lack of experimental validation of the structural changes and their direct physiological effects. Future research should incorporate in vitro electrophysiological studies, membrane modeling, and patient-derived neuronal models to validate the computational findings. Additionally, investigating other SCN9A variants, such as rs41268673 (P610T) and rs74401238 (R1110Q), and their interactions with genetic and environmental factors will be crucial in refining our understanding of Nav1.7-associated pain disorders. These insights could pave the way for targeted therapeutic strategies aimed at restoring normal pain perception in individuals with chronic pain syndromes and CIP.

## Data Availability

All data analyzed during this study are included in this article.

## References

[1] Basbaum A.I., Bautista D.M., Scherrer G., Julius D. (2009). Cellular and molecular mechanisms of pain. Cell.

[2] Breivik H., Collett B., Ventafridda V., Cohen R., Gallacher D. (2006). Survey of chronic pain in Europe: Prevalence, impact on daily life, and treatment. Eur. J. Pain.

[3] Gaskin D.J., Richard P. (2012). The economic costs of pain in the United States. J. Pain.

[4] Chaparro L.E., Furlan A.D., Deshpande A., Mailis-Gagnon A., Atlas S., Turk D.C. (2013). Opioids compared to placebo or other treatments for chronic low-back pain. Cochrane Database Syst. Rev..

[5] Dib-Hajj S.D., Geha P., Waxman S.G. (2017). Sodium channels in pain disorders: Pathophysiology and prospects for treatment. Pain.

[6] Cox J.J., Reimann F., Nicholas A.K., Thornton G., Roberts E., Springell K., Karbani G., Jafri H., Mannan J., Raashid Y. (2006). An SCN9A channelopathy causes congenital inability to experience pain. Nature.

[7] Emery E.C., Habib A.M., Cox J.J., Nicholas A.K., Gribble F.M., Woods C.G., Reimann F. (2015). Novel SCN9A mutations underlying extreme pain phenotypes: Unexpected electrophysiological and clinical phenotype correlations. J. Neurosci..

[8] Klein C.J., Wu Y., Kilfoyle D.H., Sandroni P., Davis M.D., Gavrilova R.H., Low P.A., Dyck P.J. (2013). Infrequent SCN9A mutations in congenital insensitivity to pain and erythromelalgia. J. Neurol. Neurosurg. Psychiatry.

[9] Sun J., Li L., Yang L., Duan G., Ma T., Li N., Liu Y., Yao J., Liu J.Y., Zhang X. (2020). Novel. Mol. Pain.

[10] Fertleman C.R., Baker M.D., Parker K.A., Moffatt S., Elmslie F.V., Abrahamsen B., Ostman J., Klugbauer N., Wood J.N., Gardiner R.M. (2006). SCN9A mutations in paroxysmal extreme pain disorder: Allelic variants underlie distinct channel defects and phenotypes. Neuron.

[11] Goldberg Y.P., Price N., Namdari R., Cohen C.J., Lamers M.H., Winters C., Price J., Young C.E., Verschoof H., Sherrington R. (2012). Treatment of Na(v)1.7-mediated pain in inherited erythromelalgia using a novel sodium channel blocker. Pain.

[12] Estacion M., Harty T.P., Choi J.S., Tyrrell L., Dib-Hajj S.D., Waxman S.G. (2009). A sodium channel gene SCN9A polymorphism that increases nociceptor excitability. Ann. Neurol..

[13] Reimann F., Cox J.J., Belfer I., Diatchenko L., Zaykin D.V., McHale D.P., Drenth J.P., Dai F., Wheeler J., Sanders F. (2010). Pain perception is altered by a nucleotide polymorphism in SCN9A. Proc. Natl. Acad. Sci. USA.

[14] Faber C.G., Hoeijmakers J.G., Ahn H.S., Cheng X., Han C., Choi J.S., Estacion M., Lauria G., Vanhoutte E.K., Gerrits M.M. (2012). Gain of function Naν1.7 mutations in idiopathic small fiber neuropathy. Ann. Neurol..

[15] Sereno M., Gutiérrez-Gutiérrez G., Rubio J.M., Apellániz-Ruiz M., Sánchez-Barroso L., Casado E., Falagan S., López-Gómez M., Merino M., Gómez-Raposo C. (2017). Genetic polymorphisms of SCN9A are associated with oxaliplatin-induced neuropathy. BMC Cancer.

[16] Kurzawski M., Rut M., Dziedziejko V., Safranow K., Machoy-Mokrzynska A., Drozdzik M., Bialecka M. (2018). Common Missense Variant of SCN9A Gene Is Associated with Pain Intensity in Patients with Chronic Pain from Disc Herniation. Pain Med..

[17] Leźnicka K., Pawlak M., Sawczuk M., Gasiorowska A., Leońska-Duniec A. (2023). rs6746030 Polymorphism and Pain Perception in Combat Athletes and Non-Athletes. Genes.

[18] Zhou X., Zheng W., Li Y., Pearce R., Zhang C., Bell E.W., Zhang G., Zhang Y. (2022). I-TASSER-MTD: A deep-learning-based platform for multi-domain protein structure and function prediction. Nat. Protoc..

[19] Zheng W., Zhang C., Li Y., Pearce R., Bell E.W., Zhang Y. (2021). Folding non-homologous proteins by coupling deep-learning contact maps with I-TASSER assembly simulations. Cell Rep. Methods.

[20] Yang J., Zhang Y. (2015). I-TASSER server: New development for protein structure and function predictions. Nucleic Acids Res..

[21] Zhang Y., Skolnick J. (2004). SPICKER: A clustering approach to identify near-native protein folds. J. Comput. Chem..

[22] Wiederstein M., Sippl M.J. (2007). ProSA-web: Interactive web service for the recognition of errors in three-dimensional structures of proteins. Nucleic Acids Res..

[23] Ittisoponpisan S., Islam S.A., Khanna T., Alhuzimi E., David A., Sternberg M.J.E. (2019). Can Predicted Protein 3D Structures Provide Reliable Insights into whether Missense Variants Are Disease Associated?. J. Mol. Biol..

[24] Kelley L.A., Mezulis S., Yates C.M., Wass M.N., Sternberg M.J. (2015). The Phyre2 web portal for protein modeling, prediction and analysis. Nat. Protoc..

[25] Rodrigues C.H., Pires D.E., Ascher D.B. (2018). DynaMut: Predicting the impact of mutations on protein conformation, flexibility and stability. Nucleic Acids Res..

[26] Venselaar H., Te Beek T.A., Kuipers R.K., Hekkelman M.L., Vriend G. (2010). Protein structure analysis of mutations causing inheritable diseases. An e-Science approach with life scientist friendly interfaces. BMC Bioinform..

[27] Prlić A., Down T.A., Kulesha E., Finn R.D., Kähäri A., Hubbard T.J. (2007). Integrating sequence and structural biology with DAS. BMC Bioinform..

[28] Krieger E., Koraimann G., Vriend G. (2002). Increasing the precision of comparative models with YASARA NOVA—A self-parameterizing force field. Proteins.

[29] Duan G., Xiang G., Zhang X., Yuan R., Zhan H., Qi D. (2013). A single-nucleotide polymorphism in SCN9A may decrease postoperative pain sensitivity in the general population. Anesthesiology.

[30] Holliday K.L., Thomson W., Neogi T., Felson D.T., Wang K., Wu F.C., Huhtaniemi I.T., Bartfai G., Casanueva F., Forti G. (2012). The non-synonymous SNP, R1150W, in SCN9A is not associated with chronic widespread pain susceptibility. Mol. Pain.

[31] Newton G., Charman C., Nickerson A., Phillips K., Kless A., Dunham J., Pickering A. (2025). Carriers of *SCN9A* variants linked to inherited and acquired pain syndromes show no alteration in the prevalence of pain or analgesic usage in the UK Biobank cohort. medRxiv.

